# Two Theories of Experimental Error

**DOI:** 10.6028/jres.092.016

**Published:** 1987-06-01

**Authors:** A. R. Colclough

**Affiliations:** National Physical Laboratory, Teddington, Middlesex, TW11 OLW, England

**Keywords:** error theory, experimental errors, frequency-based distributions, orthodox theory of errors, randomatic theory of errors, random errors, subjectivist distributions, systematic errors

## Abstract

The reader … will have seized my meaning if he perceives that the different situations in which uncertain inferences may be attempted admit of logical distinctions which should guide our procedure.

Sir Ronald Fisher [1]^1^

Following the widespread adoption of new approaches to the combination of experimental uncertainties, two theories of error are identified and their possible justifications assessed. They are the “orthodox theory” based on the familiar distinction between random and systematic errors and the “randomatic theory” which dispenses with the distinction and treats all errors as the orthodox theory treats random errors. The orthodox theory suffers from a number of important confusions about the nature of its central distinction, about the combination of uncertainties, and about which populations of results can correctly be said to contain random errors. These confusions are clarified and the central distinction is argued to be objective. Three justifications are developed for the randomatic theory: 1) that it is implied by the generally accepted law of error propagation, 2) that all so-called systematic errors belong to populations characterized by hitherto unnoticed frequency-based distributions, and 3) that they belong to subjectivist prior distributions. But, upon examination, the argument in terms of the law of error propagation is found to beg key controversial questions, the frequency-based distributions are found not always to be of suitable form, and the subjectivist distributions are found to be unrealistic. Thus the randomatic theory remains unjustified by objective standards. Moreover, its use could lead to the underestimation of uncertainties in the usual sense of the maximum possible or conceivable error in the result of a particular specified experiment. The concept of systematic error is argued to be indispensable and new recommendations are formulated which are orthodox in general character.

## 1. Introduction

Foundational questions in statistics are notoriously controversia1.^2^ Nowhere is this more true than in error theory which presents special problems not usually encountered in other fields of statistical practice. In particular, it often invites the experimenter, when estimating experimental uncertainties, to assess probabilities in the absence both of statistical data and the prospect of data. In view of this it is, perhaps, not surprising that confusion on the subject is widespread among experimental scientists, most of whom are specialists in fields other than statistics and unfamiliar with its foundational controversies.

Yet there is no dearth of advice about what simple procedures are to be followed in estimating uncertainties. The problem is rather that the experts disagree with one another. One reason for their disarray is of course the deeply confusing nature of the questions involved. Another is that their advice has to serve a variety of needs. Metrologists and others making fundamental physical measurements arguably require a rigorous and objective (i.e., demonstrably realistic) theory of errors on which to base accurate estimates of uncertainty. Without this it would be impossible to rule on the existence of discrepancies between the results of primary experiments. In contrast, many scientific and industrial activities require only rough-and-ready “uncertainty” estimates, and simple methods of arriving at them may be preferred to reliable ones based on objective principles. Thus the national calibration services of a number of countries employ procedures that are quite different from the traditional ones based on the distinction between random and systematic errors which most students still learn. Books, standards, codes of practice, official directives, and the like could now be cited describing them and they have much in common (e.g. [2–5]).

In recent years a great service has been done for error theory by the Bureau International des Poids et Mesures (BIPM) which has consulted very widely on the matter and produced a set of clear and concise recommendations for the estimation of experimental uncertainties that appear to be broadly in line with the widespread new procedures mentioned above [6–9]. These have provided a stimulus and a clear focus for renewed discussion of error theory which, however, remains as controversial as ever. They are set out in display box 1 for ease of reference and may be compared with the representative set of orthodox recommendations in box 2 [10].

Controversy arising from the different needs and interests of various users is probably inevitable. Its origin lies in practical considerations which are bound to be subject to value judgments of all kinds and this must preclude an uncontroversial ruling on universally appropriate procedures for the estimation of uncertainties. But, given any clear, natural, and physical definition of “experimental uncertainty” it should be possible to state unequivocally what the *correct* procedures are for its estimation even though they may not be well suited to all practical needs. The lack of consensus about what these are ought to be a matter of concern, and the purpose of this paper is to attempt to define a philosophically-neutral, physically correct and rigorous error theory without regard to practicability. In fact it is arguable that the principles to be identified for uncertainty estimation are not markedly less practicable for most purposes than are those now becoming widely adopted.

Box 1 **The BIPM’s Recommendations for the Combination Of Experimental Uncertainties**

1. The uncertainty in the result of a measurement generally consists of several components which may be grouped into two categories according to the way in which their numerical value is estimated:
  - those which are evaluated by statistical methods,
  - those which are evaluated by other means.
  There is not always a simple correspondence between the classification into categories A or B and the previously used classification into “random” and “systematic” uncertainties. The term “systematic uncertainty” can be misleading and should be avoided.
  Any detailed report of the uncertainty should consist of a complete list of the components, specifying for each the method used to obtain its numerical value.
2. The components in category A are characterized by the estimated variances, *s*_i_^2^, (or the estimated “standard deviations” *s*_i_) and the number of degrees of freedom, *v*_i_. Where appropriate, the estimated covariances should be given.
3. The components in category B should be characterized by quantities *u*_j_^2^, which may be considered as approximations to the corresponding variances, the existence of which is assumed. The quantities *u*_j_^2^ may be treated like variances and the quantities *u*_j_ like standard deviations. Where appropriate, the covariances should be treated in a similar way.
4. The combined uncertainty should be characterized by the numerical value obtained by applying the usual method for the combination of variances. The combined uncertainty and its components should be expressed in the form of “standard deviations.”
5. If, for particular applications, it is necessary to multiply the combined uncertainty by a factor to obtain an overall uncertainty, the multiplying factor used must always be stated.

The concept of uncertainty to which the following discussion relates is, in informal terms, the range within which the result of a particular specified experiment is uncertain as defined by its maximum possible or conceivable error. This entails that, when the experiment is faithfully repeated by different workers using the apparatus and procedures specified, their uncertainty bounds always or nearly always embrace the true value of the quantity to be determined and overlap each other. Moreover, when discrepancies do occur, they should always or nearly always be small compared to the uncertainties themselves. These requirements are intended to be met in a literal physical sense and uncertainty estimation procedures which do not guarantee this will be regarded as failing to implement the chosen concept. This is the concept required when judging the consistency of results. It also has the merit of not presupposing any philosophical position as would, for example, a definition in terms of standard deviations intended for application to all error types. Moreover, when “uncertainty” is defined in terms of quantities such as expected error or standard deviation, it is often because these terms are thought to give an easily calculated order-of-magnitude estimate for uncertainty as defined above and not because they express the most relevant concept themselves.

The recommendations of boxes 1 and 2 raise a number of fundamental questions, the more important being:

- What is the nature of the distinction between “random” and “systematic” quantities and how does it relate to that between Types “A” and “B”?
- Is it objective and useful or merely a distinction without a significant difference?
- Is it legitimate to represent *all* uncertainties, including those evaluated by other-than-statistical means, by statistical or quasi-statistical quantities?
- Is it legitimate to combine uncertainties of different types as though combining variances of random variables of zero mean?

Box 2 **Representative Orthodox Recommendations for the Combination Of Experimental Uncertainties [10]**

The uncertainty on a measurement should be put into one of two categories depending on how the uncertainty is derived: a random uncertainty is derived by a statistical analysis of repeated measurement while a systematic uncertainty is estimated by nonstatistical methods.

When combining the uncertainties on individual measurements in a complex experiment involving measurements on several physical quantities the two categories of uncertainties should be kept separate throughout.

In such an experiment the total random uncertainty should be obtained from the combination of the variances of the means of the individual measurements together with those associated with any constants, calibration factors, etc.

The component systematic uncertainties should be estimated in the form of maximum values or overall limits to the uncertainties.

In reporting measurements of the highest accuracy, a full statement of the result of an experiment should be in three parts, the mean corrected value, the random uncertainty, and the systematic uncertainty … The components that have contributed to the final uncertainty should be listed in sufficient detail to make it clear whether they would remain constant if the experiment were repeated … The estimate of the total systematic uncertainty should be stated … Each component of the systematic uncertainty should be listed, expressed as the estimated maximum value of that uncertainty … The method used to combine these component (systematic) uncertainties should be made clear.

The combination of random and systematic uncertainties to give an “overall uncertainty” is deprecated, but if in a particular case this is thought to be appropriate then it should be given in addition to the two uncertainties, together with the method of combination.

Different answers to these questions will be obtained depending on the general theory of errors that is adopted and how the various concepts referred to are defined within it. Two general theories will be identified below, clearly formulated and used to derive answers to these and related questions. They will be referred to as the “orthodox” theory which retains the distinction between random and systematic quantities and the “randomatic” theory which dispenses with it and treats all errors and uncertainties on an equal footing. It is on the latter theory that the BIPM recommendations appear to be based.

In formulating the two theories, it will be necessary to be clear about the objectivity of various error populations to be considered and the probability distributions to be defined over them. No serious controversy need arise about the physical status of populations of results and their corresponding errors when they are produced by repeatable measurements according to a well-defined experimental specification that permits random variations about nominal conditions. Nor is there a problem about errors which are sampled at random in some physical sense from a pre-existing error population (perhaps when an instrument with a certain zero error is chosen from a population of similar instruments in which a corresponding population of zero errors exists). But the objectivity of populations cannot be countenanced when an experimental specification is too loose to produce properly controlled results or when pre-existing populations are not unambiguously identified.

All schools of philosophy accept the notion that probability *evalualions* based on the frequencies observable in an objective statistical population are themselves objective. This is true irrespective of the particular view held of the concept of probability itself (for example, that it is a long-run frequency or a subjective “degree of belier’). Thus frequency-based probability evaluations are philosophically neutral and so unproblematic in error theory as in other fields of statistics.

But there is one school of statistical thought and practice of particular relevance to error theory where probability evaluations that are not frequency-based are employed freely with those that are. In subjectivist statistical method a “prior probability distribution” describing a subject’s “degrees of belier” in the various possible outcomes of some trial *before* results are obtained is estimated, perhaps in a vague way on the basis of general experience. When statistical data are subsequently gathered this prior probability distribution is “conditionalized” by the application of Bayes’ theorem to produce a frequency-based posterior distribution which, given sufficient data and not-too-wild a choice of prior distribution, agrees closely with that obtained by other statisticians (see section 3.3). If the subjectivist’s prior degrees of belief are based on correct, but approximate, physical information, his prior distribution will be approximately physically objective as well as being, presumably, psychologically objective. If, on the other hand, it is a mere unfounded intuition or a guess, it will not generally be physically objective. Nor will it, if, in acknowledgment of ignorance, the subjectivist assigns equal probabilities to all possible values because he has no reason to prefer one to another (Bayes’ postulate). Where the latter types of probability evaluation are employed in error theory, there will be a serious question about the objectivity of uncertainty evaluations calculated from them.

## 2. The Orthodox Theory of Errors

Although orthodox error theory is characterized by a central distinction between “random” and “systematic” errors, the exact definitions of these key terms are vague, and confusion exists about what methods of combination are correct for the corresponding types of uncertainty and about the correct classification of error types arising from various causes. A clearer statement of the orthodox theory will therefore be formulated with which most orthodox error theorists would be in general agreement. Those adhering to it will be referred to, purely for convenience and in a narrow sense only, as “conservatives.”

### 2.1 The Formulation of the Orthodox Distinction Between Systematic and Random Uncertainties

There are three possible approaches to the classification of errors as systematic or random. Definitions may be cast in terms of …

1. how they would behave if an experiment were repeated (e.g., in terms of the forms which their distributions would take),
2. how their causes would behave upon repetition of the experiment or the nature of their causes (e.g., scale errors, rounding, fluctuations of one kind or another, mistakes), and
3. the way they are treated (e.g., by statistical means or on the basis of a theoretical estimate).

Confusion often arises in elementary accounts of the nature of errors because these various approaches are not clearly distinguished. In this section the classification of errors will be based initially on their behavior when an experiment or, perhaps, some associated “trial,” is repeated [approach 1) above]. The combination of uncertainties will be dealt with mainly in the following section. The important practical question of how error types as defined by their behavior are to be identified in terms of their known causes will be discussed in section 2.3. In the interest of brevity, the term “experiment” will stand in what follows either for a single measurement; a set of measurements, some of which may be repetitions; or for a whole experiment as usually understood. The term “result” will be used for the value obtained from an experiment in any of these three senses.

#### The Fundamental Four-Fold Error Classification

**Figure 1:**
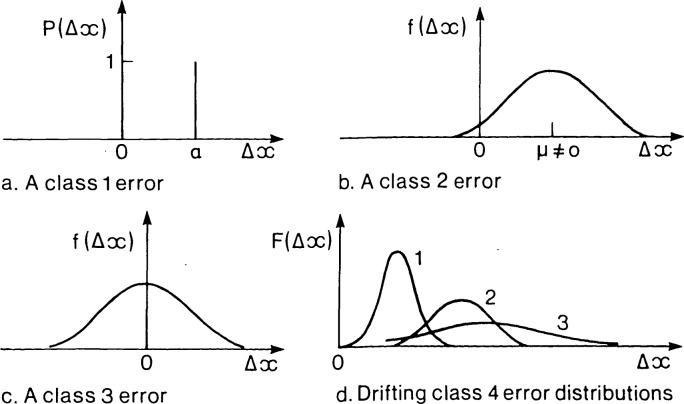
The four classes of error defined in the text.

When an experiment is repeated many times, four types of behavior are possible in the observed results as shown in figure 1 …

1. each result may differ from the true value by the same amount and with the same sign, i.e. the error is constant,
2. each error may vary randomly realizing a stable random distribution with a non-zero mean,
3. each error may vary randomly realizing a stable distribution with a zero mean, or
4. each error may vary non-randomly (e.g., cyclically or by failing to produce convergent frequencies).

These four classes of error are doubtless capable of further division, but the classification as it stands is obviously unique in any given case and exhaustive since it consists of successive *dichotomies* or disjunctions of logical complements: constant error or varying error (non-randomly or randomly (nonzero mean or zero mean». In other words, *there are no errors which do not belong to one or another of these four classes and none belonging to more than one.* Since the classification is exhaustive, any other classification of error-related concepts, including that in terms of systematic and random types, must embrace all four classes if it is also to be complete.

#### The Definition of ‘Random’ and ‘Systematic’ Errors

Although the exact nature of the distinction between systematic and random errors is often a matter of confusion, the practical motive behind it is clear enough. It arises from the perception that some errors, the “random” ones, can be treated statistically and in principle reduced to any desired level *solely on the basis of results*, while others, because of a tendency to act in one particular direction, cannot. The latter group of errors, the “systematic” ones, must therefore be assessed, and perhaps corrected for, independently of results.

But however clear the motivating ideas may appear, there is a widespread and crucial confusion in orthodox error theory about what types of population of results may be said to contain random errors. Must the results be *actually observed* by the experimenter when repeating his experiment before the existence of random errors can be contemplated? Or is it sufficient that the results *could be* observed repeatedly, though the experimenter chooses to conduct a measurement just once? Can errors in the repeatedly observed or single results of others be regarded as random when the results are used to calculate that of one’s own experiment? Could it ever be correct to regard the error in the single result of some “trial” associated with an experiment, but not usually regarded by the conservative as part of it, as random (e.g., a scale error in an instrument “randomly” chosen for use)? These questions will be addressed later, but here definitions of “random” and “systematic” will be formulated which could be applied to any population of results accepted as “statistical.” For simplicity the initial discussion is cast in terms of results obtainable by repeating an experiment.

Clearly a class 1 error could never be evaluated by contemplating a sample of results, however large, since, being the same for each result, it would lead to no differences in successive values from which its magnitude and sign could be inferred. An error of this kind, caused perhaps by a constant unwanted and uncorrected physical effect, is often regarded by the conservative as the standard case of a systematic error. As such, it is contrasted with a class 3 error which can be assessed in detail and reduced to any desired level by taking the average of a sufficiently large sample of results. This is the standard case of a random error.

The relation of class 2 errors to the random versus systematic distinction is less straightforward. The conservative frequently likes to oppose systematic to random errors, yet here is a randomly distributed error which nevertheless introduces on average a non-zero error into results which cannot be reduced indefinitely solely by averaging a large sample. However, while it may not be obvious how to classify class 2 errors themselves, every class 2 error can clearly be said to consist of a class 1 systematic component and a class 3 random one, the former component being identified with its mean or expected value.^3^ Indeed, since the evaluation and treatment of uncertainties is always carried out separately for each component, there is no practical necessity to classify class 2 errors themselves. Definitions of “systematic” and “random” might therefore be adopted which result in class 2 errors being one, the other, neither, or both according to taste.

The above view of the mixed composition of class 2 errors need not, of course, imply an assumption that their constant or systematic component corresponds to any single physical cause or group of causes different from those giving rise to the random variation. Although they can be produced by distinct class 1 and class 3 errors, generally both components will have a cause or causes in common and in that sense are part of the same error. For this reason formal definitions of “random” and “systematic” would need to refer both to errors and error components.

Cast in terms of a result *y* instead of an error Δ*y* ≡ *y – y*_0_ where *y*_0_ is the true result, the definition of systematic errors as class 1 errors or error components is equivalent to that sometimes offered in terms of statistical bias: *E (y*) − *y*_0_.^4^

Class 4 errors are probably far more common than is generally realized. For example, any error that increases uniformly with time, even if “sampled” at random intervals, would be of this kind. In spite of this their existence is not usually recognized. Class 4 errors cannot, of course, be counted as random, but it is of little practical importance whether they are held to be systematic or are neither random nor systematic.

In the light of these considerations “systematic” and “random” errors might be defined by the scheme set out in box 3 or by equivalent definitions which would not necessarily be cast in terms of the four-fold error classification. Class 2 errors, the categorization of which was seen to have no practical significance, have been arbitrarily taken to be neither random nor systematic and class 4 errors to be systematic.

The definitions of errors of classes 1 to 4 were physical ones cast in terms of what behavior would be observed if an experiment were repeated many times according to a clear experimental specification. Thus what class an error belongs to is a completely objective matter when it arises in results of repeatable measurements. Since the definitions of “random” and “systematic” error of box 3 are cast in terms of error classes 1 to 4, they too are objective categories applicable to all such errors.

Box 3 **A Possible Definition of ‘Random’ and ‘Systematic’ Errors**

**Table:** 

Orthodox Category	Error or Error Component
Systematic	class 1 error
	class 1 component of class 2 error
	class 4 error
Random	class 3 error
	class 3 component of class 2 error
Neither	class 2 error

It should also be noted that the subjunctive or “counterfactual” nature of the definitions (“… *would* be … if an experiment *were* …”) enables single-reading errors to be called “random” or “systematic” even though the concepts are defined relative to a large population of errors. This should not be a matter for concern, of course; physical properties are typically “dispositional” in this way. That is, they are manifested only under appropriate conditions, but are held to persist in their absence. This important point will be discussed further in section 2.3.

#### The Definition of ‘Systematic Uncertainty’

Once “systematic error” is defined, “systematic uncertainty” can be defined in terms of it. There are several ways of doing this of which the simplest is the following:

The “systematic uncertainty” in a given direction in the result of an experiment is *the magnitude of the range of its possible values as defined by knowledge of its maximum possible systematic error or error component in that direction.*

This concept of systematic uncertainty has been criticized because limits which are literally the maximum possible are often absurdly large and in most practical cases there is an ineliminable element of “subjective” judgment in assessing plausible ones. Indeed, it is at this point that some experimenters abandon orthodoxy and introduce probability concepts to confine the range of the error to lie within conceivable rather than possible limits (cf. the definition of “random uncertainty” below).

But the conservative does not concede that it is appropriate to treat all errors as random errors. He usually prefers to abandon the definition of “systematic uncertainty” in terms of maximum possible error, but maintains that there are systematic errors which are not randomly distributed in *his* experiment (e.g., errors due to the use of biased theoretical corrections required by its specification). Uncertainty is therefore to be treated in terms of what Eisenhart has called “credible bounds” [11]. These are often said typically to be less than the maximum possible bounds, but if probabilities are employed in judging them they are held not to contribute to the random uncertainty of the final result. The conservative may also wish to maintain that there are some practical cases where admissible probabilistic information is lacking and where credible bounds are best replaced by maximum possible bounds.

#### The Definition of ‘Random Uncertainty’

The expression “random uncertainty” is conventionally defined in terms of “random error” as follows:

The “random uncertainty” in a given direction in the result of an experiment is *the magnitude of the range of its values as defined by a knowledge of its maximum conceivable random error or error component in that direction.*

The use of “conceivable” here where “possible” was used in the previous definition, is in recognition of the common necessity of choosing a confidence level of less than 100% probability which for many distributions corresponds to the range ± infinity. The justification of this procedure, apart from necessity, is that everyone is prepared to discount possible exceptions at some low level of probability.

### 2.2 The Orthodox View of the Combination Uncertainties

The law of error propagation states how various errors in an experiment combine to produce the error in its final result. Unfortunately the combining errors are not usually known, else they could be corrected for at source. What are usually known instead are their estimated maximum possible values, credible bounds, variances, or other quantities related to their respective uncertainties. How does the conservative use this information to estimate the uncertainty of his final result?

#### Orthodox ‘Combination’ of Random Uncertainties

The estimation of the random uncertainty resulting from the combined effect of two independent random errors is unproblematic in principle. The distributions of the errors convolute and their standard deviations combine in quadrature (i.e., their variances add) to produce those of the resultant error. Resultant random uncertainty is to be estimated from the resultant distribution relative to some choice of a confidence level close to one. It should be noted that in general random uncertainties, as opposed to standard deviations, do not combine in quadrature to yield a correct resultant random uncertainty. This may easily be demonstrated by consideration of the combination of two similar, but independent, uniformly distributed random errors, for example, which yields a resultant with a triangular distribution. The only exception to this rule arises from the combination of normally distributed errors which interact to form another normally distributed error; here uncertainties do combine in quadrature. But in general, unlike the expression “combination of errors,” the phrase “combination of uncertainties” can be misleading.

#### Orthodox Combination of Systematic Uncertainties

Wavering conservatives sometimes entertain the notion that systematic uncertainties can be combined in quadrature to obtain a resultant systematic uncertainty [10, 11]. This view may arise from feelings that it would be improbable that many systematic errors would all pull in the same direction or, more specifically, that *p*(+)=*p*(−)=0.5 (Bayes’ postulate applied to signs); that in ignorance of their values they are uniformly distributed between bounds (Bayes’ postulate applied to errors); and that credible bounds must be something like standard deviations because they are assessed from probabilistic considerations. However, combination in quadrature of systematic uncertainties is fundamentally inconsistent with the orthodox theory one of the first principles of which is that there exist constant errors and error components. That constant *error*-like quantities combine in a linear way is accepted by everyone. There is no dispute that when the distributions of combining random variables are convoluted to produce a resultant distribution, the mean of this distribution is simply the arithmetical sum of those of the combining variables. This is true in error theory as in other fields of statistics and applies in particular to class 2 errors. To the consistent conservative the rationale for this is that the means are to be regarded not as random variables but as constants of the experiment of unknown sign and magnitude (or “constants of nature” in general statistical parlance). This is because they are parameters of *particular* error populations explicitly or implicitly identified by any complete experimental specification. As such, the means cannot be said in any physical sense to be drawn from a population and are undistributed except, perhaps, in the form of a delta function at some unknown location between credible bounds. Since no probabilities can be assigned to their various possible values the upper limit to be placed on the sum of the means can only be obtained from the sum of their individual upper limits, however defined. This becomes a simple point of logic where upper limits are defined to be maximum possible values. But even when credible bounds are employed, they are still intended to confine the conceivable values of unknown, undistributed constants which are agreed to combine in a linear way and could all pull in the same direction. Thus the consistent conservative permits himself no recourse to statistical procedures in such cases and must recommend that systematic uncertainties be combined in a linear way. Even if it were thought that systematic quantities were randomly distributed, uncertainties as opposed to systematic “standard deviations” would not be the appropriate quantities to combine in quadrature as argued above (cf. box 1, recommendation 3).

#### Orthodox Combination of Random With Systematic Uncertainties

**Figure 2:**
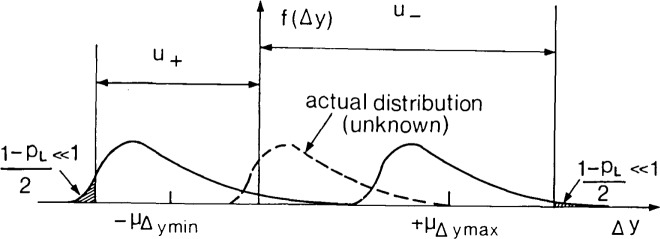
The orthodox method of calculating uncertainties corresponding to class 2 errors applying a confidence level to two worst-case distributions.

How are uncertainties corresponding to “mixed” (class 2) errors to be evaluated on the orthodox view? Little guidance on this important matter is to be found in conservative literature, but a procedure is easily devised. In the case of a class 3 error, uncertainties *u*_+_ and *u*_−_ are obtained from a confidence level *p*_L_ applied to a *single* class 3 distribution. In calculating *u*_+_ and *u*_−_ for a class 2 error, the consistent conservative must consider not one distribution, but *two* different worst-case distributions as shown in figure 2. These arise in the following way:

– the form of the distribution of the purely random component of the error is observed or inferred as it might be for the case·of a class 3 error, but its mean μ_Δ_*_y_* is unknown,
– the maximum positive limit on its mean, μ_Δ_*_y_*_max_, is obtained by summing its component limits in the way argued for above,
– similarly, a minimum negative limit on its mean, − μ_Δ_*_y_*_mim_ is calculated,
– one worst-case distribution is obtained by setting μ_Δ_*_y_* = + μ_Δ_*_y_*_max_ simply because this represents one of the two worst conceivable situations.
– similarly, the other worst-case distribution is obtained by setting μ_Δ_*_y_* = − μ_Δ_*_y_*_min_.

The probable presence of large positive errors implies the necessity of a large negative uncertainty. Thus in order to obtain a value for *U*_−_, the conservative now “slides” a vertical boundary out along the positive error axis until a small fraction (1−*p*_L_)/2 of error values is enclosed beneath the curve to the right of the line. A similar process conducted in the opposite direction will yield *u*_+_. (The convention of choosing a value of *p*_L_ to exclude a fraction (1−*p*_L_)/2 rather than (1−*p*_L_) ensures continuity with the usual convention for class 3 errors as μ_Δ_*_y_*_max_ and μ_Δ_*_y_*_min_ both approach zero.)

This procedure covers cases where positive and negative systematic uncertainties or the random components of errors or both are disposed asymmetrically. But it does not, of course, allow probabilities to be associated with *u*_+_ and *u*_−_ as with the uncertainties corresponding to class 3 errors because none was associated with μ_Δ_*_y_*_max_ and μ_Δ_*_y_*_min_. It might be said that *at least* an estimated fraction *p*_L_ of the results of a repeated experiment would lie between *y* − *u*_−_ and *y* + *u_+_*, but not that an estimated fraction 1 − *p*_L_ would lie outside this range. For this reason no probabilities can be associated with the compatibility of two experimental results *y*_a_ and *y*_b_(>*y*_a_) where either or both have mixed errors. They agree if *u*_+a_+*u_−_*_b_*<y*_b_−*y*_a_. If *u*_+a_*+u*_−b_<*y*_b_−*y*_a_, they disagree. On the orthodox view, there is no more to be said.

No analogous analysis of error-related quantities other than uncertainty is offered here. The estimation of expected values of errors, of their expected absolute values or of rms values requires that μ_Δ_*_y_ or E*(μ_Δ_*_y_*) is known. The conservative believes that they are equal because the former is a constant and not a distributed random variable. But since it is an unknown constant he is bound to regard the derivation of expressions for expectations to be of no practical use. It will be seen later that supporters of the randomatic theory take a different view and that *E*(μ_Δ_*_y_*) is assumed to be calculable even when μ_Δ_*_y_* is not known with certainty.

### 2.3 Error Types and Their Identification on the Orthodox View

It has already been noted that there is often confusion about what populations can legitimately be said to contain random errors for the purpose of estimating uncertainties in experimental results. Since the “combination” of uncertainties depends on the identification of the corresponding error types, this is a matter of some practical importance and the confusion needs to be resolved.

At the beginning of section 2.1, it was pointed out that the distinction between systematic and random errors was sometimes based upon error causes [approach 2)], rather than upon the behavior of errors as experiments were repeated [approach 1)]. It will be clear that equivalent definitions of “systematic” and “random” could be cast in terms of causes, provided allowance was made for any nonlinear dependence upon them of the resulting errors.

While the above possibility is widely intuited, it has never been developed to the author’s knowledge. It nevertheless seems tacitly to underpin a different and much less satisfactory type of exercise intended to define systematic errors in terms of their causes. Here systematic errors in results are defined by an enumeration of systematic causes. Some typical cases are shown in box 4. But such enumerations amount only to definition by example and so cannot be fundamental. Without a prior criterion stating how causes are to be related to one category or the other, the classification would not be possible. In the absence of a statement of the criterion the procedure remains obscure and, equally important, there is no way of telling if it is complete. It seems clear, however, that in each case the vague underlying notion is that such errors are class 1 or constant errors. Unfortunately the errors listed in box 4 do not always behave as class 1 errors when the *relevant* trial is repeated, as will be argued below. The enumeration is based on simplistic rules-of-thumb which are no substitute for a physical analysis of the way their causes operate. In what follows, errors defined in this way will be referred to as “so-called” systematic errors to distinguish them from those defined in terms of their behavior (cf. box 3).

Also mentioned at the beginning of section 2.1 were definitions of “systematic” and “random” cast in terms of how errors or uncertainties are actually estimated or treated in a given case, rather than in terms of what is possible or proper in view of their nature [approach 3)]. When such definitions are offered it is sometimes unclear whether it is intended that the method of evaluation or treatment determines what category errors fall under or *vice versa.* Here it will be assumed in the interest of objectivity that it is the nature of the error which determines the correct method of evaluating its corresponding uncertainty, so that no definition purporting to be fundamental need ever mention the actual methods of evaluation employed in given cases. The opposite view is again related to the aforementioned confusion about which populations of results can be said to contain random errors. The errors of box 4 frequently arise from random processes and so arguably contain a random component. But such components will have a constant effect when combining with errors in the results of other measurements, no matter how often, the latter are repeated. From this it is sometimes concluded that the errors of box 4 cannot be held to contain a random component for the purpose of calculating an uncertainty in one’s own result. Thus one author, having given examples of systematic errors, writes, “There is no strict definition of systematic errors, since what is systematic for one experiment may not be for another” [12]. Another states, “One has to remember that some errors are random for one person and systematic for another” [13]. This outlook may have led to the mistaken view that the central orthodox distinction is mutable and that the labeling of errors as “random” or “systematic” is somehow conventional. Credence has thereby been lent to the notion that what is actually done is as important as why it is done. Fortunately the confusion can be removed by resolving certain more fundamental ones about probabilities and it can be stated definitively what populations can correctly be said to contain random errors. It will then be clear that the distinction between random and systematic errors is an objective one and that the nature of any given error is fully determined once a complete experimental specification has been formulated.

Box 4 **An Orthodox Definition of ‘Systematic Error’ By Enumeration of Causes (‘So-called’ Systematic Errors)**

“Systematic errors” are those owing to:

- Single readings
  – rounded
  – interpolated
- Instrument errors
  – calibration errors
  – other scale errors
  – errors due to “subclinical” malfunction
  – errors due to bad practice
- Residual correction errors arising from inexactness in correcting for known systematic effects
- External errors arising from results taken from other experiments

The confusion is well illustrated by the much-discussed problem case of external errors. Two possibilities exist for their treatment. The error in an external quantity might be taken to be entirely systematic, even where the worker producing the result can be said to have correctly assessed it as being entirely random or part random and part systematic. The alternative is to take over the error assessment, assumed correct, of the original worker in deriving the uncertainty of one’s own result. The justification of the former view is that no matter how often the main experiment is repeated, errors in the external result will always affect the answer in the same direction and to the same degree; i.e., it is a class 1 error. Thus one experimenter’s random error is another’s systematic error. But the opposing view notes that if the external measurement had been conducted by the “borrower,” it would be regarded as an ancillary measurement in his own experiment and no question would arise of changing any random component in its uncertainty to a systematic component. Who did what is held to be an unphysical consideration which could not change the nature of an error and so the original worker’s analysis is to be retained.

The position will be adopted here that the latter argument is the correct one. While the random component of an external error certainly will affect the result of the experiment in hand with a definite sign and magnitude (what other way is there?), this is true only in the sense that it is true for its own internal random error. The best estimate of the internal error introduced by an external random error component is zero with an uncertainty based on the latter’s distribution (cf. Campion et al., [14]).

To see this more clearly some may find it helpful to consider a simple gaming analogy of a class 3 error. Suppose a die, possibly loaded, is thrown repeatedly to estimate the unknown expected value μ*_n_* ≡ *E* (*n*) of the outcome *n* (1 to 6). Here μ*_n_* is analogous to a true value to be determined by measurements, *n* to the observed (digital) results, and *n* − μ*_n_* to a class 3 error. After a single throw the “error” *n* − μ*_n_* is, like a measurement error in a single reading, physically determined, but unknown. Nevertheless, everyone would accept that some unknown, but objective, probability was in principle to be associated with it and that this would be the same whether one threw the die oneself or someone else threw it. For example, in the case of a fair die *p*(*n−*μ*_n_*)= 1/6 for any *n* where μ*_n_*=21/6.

Suppose further that it is desired to assess the uncertainty in an estimate $\bar{n}$ of μ*_n_* obtained as the mean of 100 outcomes, one of which was provided by an outsider. Would it be correct to calculate random uncertainties ±u in Σ*n*/100 at, say, the 95% level of confidence for 99 throws and then to augment these by maximum error limits, $u_{+}=(\bar{n}-1)/100$ and $u=(6-\bar{n})/100$, corresponding to the single “external” result? Of course no one would proceed in such a way. The uncertainties would be calculated at the 95% level of confidence for the full 100 throws. Thus “external” random quantities are to be treated no differently from “internal” ones as asserted.

This justification for the ruling that external random errors are not to be distinguished from internal ones is easily generalized and so provides a basis for the resolution of the question of which populations of results can legitimately be said to contain random errors. It means that in principle *any* error determined by a random process, whether under the management of the experimenter or not, is to be treated as a random or part-random error even if it occurs in a single result. This accords with universally accepted statistical principles as applied in the above gaming example and licenses experimenters to treat many so-called systematic errors, or components of them, as random. Thus random errors in the result of an experiment can arise from external results, from calibrations and, if an experimenter’s instrument can realistically be said to be sampled at random from some population, from instrumental imperfections which do not change in the course of his measurements. Similarly, if a conservative were, unusually, in a position to assess “credible bounds” for a so-called systematic error in his experiment using knowledge of the form of its random component, these bounds too should be treated as partly or wholly random uncertainties. As a result of these reforms of orthodox practice, reductions can be made in many overall experimental uncertainties conservatively estimated on the incorrect assumption that some of their components were purely systematic. This is so because combination in quadrature is permitted for the standard deviations of the newly identified random error distributions. However, as will become clear in section 3, the fact that all assessable external random error components are to be treated as such does not imply that all external error components are random.

From these considerations it is clear that the identification of an error as random or systematic or a mixture of both should be based on an analysis of the way its causes would operate upon repetition of the experiment or some associated trial. In particular it is necessary to identify all random mechanisms which can affect it even though they are’ normally regarded as being outside the experiment. The correct identification of error types will be ensured if attention is directed to the *whole* experiment including those parts conducted by others and the random trials performed, perhaps unwittingly, by oneself (for example, the choice of an instrument). Each repeatable operation in the whole experiment, whether actually repeated or executed just once, should have exactly the same status as one’s own repeated measurements. This broad and rational outlook may be contrasted with the uncritical use of rules of thumb such as those illustrated in box 4.

Six common related failings of conservative argument and practice have been encountered in this section. To summarize, they are …

– vagueness about the meaning and objectivity of the basic distinction between random and systematic quantities,
– a confusion about scope: which populations can be said to contain random errors?
– vagueness about the correct method of combining systematic uncertainties.
– vagueness about the correct method of calculating uncertainties corresponding to mixed (class 2) errors.
– the misidentification of error types by the naive use of rules of thumb, and
– failure to notice the widespread existence of random errors.

The realization that the role of random errors in experiments is much wider than orthodox assumptions sometimes allow has doubtless been a stimulus to the alternative view offered by the randomatic theory. And the fact that conservatives appear to have forgotten the reasons for orthodox practice has made the task of the randomatic theory’s proponents an easier one.

## 3. The Randomatic Theory of Errors

To those nurtured on the orthodox view, the randomatic theory of errors seems initially to be very radical. As the first main tenet of the theory, the distinction between random and systematic errors is held either to be a merely conventional distinction without an objective difference or to be a real, but irrelevant, distinction for the purposes of determining uncertainties in practical cases. However, which of these views is held by any given proponent of the randomatic theory (or “randomaticist,” for ease of reference) may not always be clear. The second main tenet of the theory is that all uncertainties are to be calculated by statistical techniques, for example by combining “standard deviations” in quadrature irrespective of how a conservative error theorist would classify their corresponding errors.

There can only be three main types of justification of the new theory. The first, presented in section 3.1, takes as its starting point the generally agreed law of error propagation and uses it to attempt to show that random and systematic standard deviations, so-called, are logically required to be combined in quadrature [15–17]. That being the case, the distinction, whether originally valid or not, is shown to be irrelevant for arriving at an overall assessment of uncertainty. On this approach, the randomaticist’s second tenet would appear to be the more fundamental.

The second and third justifications of randomatic procedures depend not on the law of error propagation, but on the assumption or perception (depending on one’s position) that all errors of interest classified by the conservative as systematic can be associated with random variables having a parent population over which a probability distribution can be defined. The conservative, on this view, has simply failed to notice something useful; namely that all errors are random and can, even according to his own beliefs, be treated statistically. It is thus implied that the central conservative distinction corresponds to no real difference and the first randomatic tenet plays the more fundamental role by providing a justification for the second. This type of argument can be fundamentally different when cast in frequency-based terms (Justification 2 presented in section 3.2) from that cast in subjectivist terms (Justification 3 presented in section 3.3). A number of authors have proposed procedures for uncertainty estimation based on the assumption that all errors can be represented by random variables, but it is generally unclear whether Justification 2 or 3 is intended [18–21].

### 3.1 Justification 1: Randomatic Theory via the Law of Error Propagation

The law of error propagation, which is quite uncontroversial, states that the error Δ*y* in the result *y* of an experiment is given by

$$\Delta y=\sum (\partial y/\partial x_{j})\Delta x_{j}$$

where the Δ*x_j_* are the errors in the various individual or repeated measured values of *x_j_* of the experiment. To illustrate why it is thought that both random and systematic “standard deviations” are to be combined in quadrature the simple case will be considered where the required result of an experiment is the mean of *n* similar results *x_j_: y* = Σ*x_j_/n.* Let the *x_j_* suffer systematic errors *+a* and *×b* with a random error *R_j_* so that Δ*x_j_=a +*(*b* − 1)*x_j_+R_j_.* Approximate corrections −(*a* − Δ*a*) and 7(*b* − Δ*b*) would generally be made to the observed *x_j_* whereupon Δ*x_j_*=Δ*a* + Δ*bx_j_* + *R_j_*. It is easily shown that, *provided* the expectation *E*(Δ*a*Δ*bx_j_*)=0, the law of error propagation implies:

$$E(\Delta y^{2})=\Delta a^{2}+\Delta b^{2}{\bar{x}}^{2}+{\sigma_{x}}^{2}/n$$

where ${\sigma_{x}}^{2}\equiv E({R_{j}}^{2})$ i.e., “standard deviations” of residual correction errors and random errors combine in quadrature. It is assumed that in any well-designed experiment significant systematic errors will always be corrected for and that this therefore provides a general rationale for practical randomatic procedures of the kind which those recommended in box 1 appear to be.

#### Problems with Justification 1

The conservative, is unlikely to find Justification 1 convincing for two reasons. Firstly he would not accept that systematic errors were always corrected for in well-designed experiments. There are many cases where a systematic error is tolerably small and where a reliable correction is difficult to estimate. Here the experimenter will often prefer to leave it uncorrected and to estimate the uncertainty in terms of bounds. For the argument to work in such a case it would be necessary to assume not that *E*(Δ*a*Δ*bx_j_*)=0, but that *E*(*a*(*b−*1)*x_j_*)=0, which is only true in general if the errors *+a* and *+*(*b*−l)*x_j_* are uncorrelated class 3 ones like the *R_j_.* But this is exactly what the conservative denies; it will be recalled that the existence of class 1 errors is the first principle of his theory. If anyone repeating the experiment according to the same experimental specification could be expected to encounter the same constant values of *a* and *b*, then it would be the case that *E*(*a*(*b*−1)*x_j_*)=*a*(*b*−1)*x_j_*. More generally, if the errors *+a* and ×*b* were always drawn from the same two respective populations with unknown non-zero means μ*_a_* and μ*_b_*, then $E[a(b-1)x_{j}]=\mu_{a}(\mu_{b}-1)\bar{x}\neq 0$.

The conservative’s second objection would be that, even where corrections are made for systematic errors, different residual correction errors are not generally statistically independent class 3 errors either. For example if *a* and *b* arose from two corrections made for systematic effects on the basis of simplified theoretical models which all experimenters following the specification would be expected to use, then Δ*a* and Δ*_b_* could be constant class 1 errors in which case $E(\Delta a\Delta bx_{j})=\Delta a\Delta b\bar{x}$. From this it follows that

$$E(\Delta y^{2})={\left(\Delta a+\Delta b\bar{x}\right)}^{2}+{\sigma_{x}}^{2}/n$$

which is the usual conservative formula with systematic errors combining together in a linear way. The enlightened conservative believes that typically residual correction errors, like most so-called systematic errors, would be of class 2 so that $E(\Delta a\Delta bx_{j})=\mu_{\Delta a}\mu_{\Delta b}\bar{x}\neq 0$ as before. It is therefore the case that Justification 1, though perfectly correct given certain randomatic presuppositions, cannot be used to establish those presuppositions on pain of circularity. The conservative will see the argument as begging certain key questions as controversial as Tenet 2 itself. The same applies to any justification employing a statistical proof that standard deviations combine in quadrature and which implicitly assumes that all errors are random variables of zero mean (e.g., [22]). It will become clear that Justification 1 is implicitly dependent on the reliability of Justification 2 or 3.

### 3.2 Justification 2: Randomatic Theory via Frequency-Based Statistical Distributions

This justification depends upon the assumption that every systematic error belongs to a well-defined stable population which can be generated by repeated measurements or by some other repeatable trial associated with the experiment. For example a barometer zero error might be said to be long to and be sampled from the population of zero errors realizable by constructing an infinite population of barometers to the same engineering specification and perhaps subjecting them to the same calibration procedure. The error would thus be fixed for any given experimenter executing the experimental procedure, but would be a random variable analogous to a single reading (cf. the discussion of conservative attitudes to such quantities in section 2.3). From this it might be argued that all errors were random errors.

#### Problems with Justification 2

The results of measurements repeated according to a clear experimental specification, and the corresponding errors, belong to well-identified populations; those defined in advance by the specification. But what population do those systematic errors “outside” the experiment belong to? It might be argued that if a systematic error could be assigned to more than one population equally naturally with no means of identifying the “right” one, different but equally correct standard deviations and uncertainties could be derived. They could not therefore be objective quantities (cf. Ayer [23]). For example, does a barometer zero-error belong to 1) the population of zero-errors realized by repeated constructions to the same specification or to 2) the different population of zero-errors to be found in barometers available for use in (say) British laboratories? Since randomaticists do not identify their populations, but simply invoke distributions or even just standard deviations, their calculated uncertainties cannot in practice be objective frequency-based ones.

However, though this may be true of informal practice, there is no deep problem of principle here for the randomatic theory. So-called systematic errors really can belong to several natural populations from which they are simultaneously sampled. The experimenter may use his approximate knowledge of these to choose or define the population characterized by the smallest errors as the basis of his calculation of uncertainties, provided that the population involved really would be randomly sampled by repetition of the error selection procedure actually employed in his experiment (e.g., through the purchase of a barometer by his organization). If, for example, he judges that zero errors of British barometers in general would only very rarely exceed ±30 Pa, but that his particular design would limit this to ± 10 Pa, then it is legitimate to use the latter information ignoring the former. Different experimenters may draw their barometers (and their zero errors) from the same or different populations. But if these are properly identified and their corresponding distributions or bounds plausibly assessed, uncertainties will be correctly estimated in each case. Because the population sampled is a determinant of the experimental result and its error, it will be supposed in all that follows that it must be explicitly or implicitly identified in a complete experimental specification and is not to be regarded as a matter “outside” the experiment (cf. section 2.3).

But there is a more serious objection to Justification 2 than the charge that randomatic populations of systematic errors are not uniquely identifiable. This states that their distributions are not generally of class 3 having zero means. As noted in the previous section, unknown non-zero means for residual correction errors are only to be expected. And this is true in general of the frequency-based distributions characterizing genuinely physical error populations. For example, there is no reason to suppose that the populations 1) or 2) above have zero means. Indeed there exist many errors which can only have one particular sign and for which corrections are not made. Thus if randomatic procedures are to be justified, it cannot be in terms of frequency-based distributions.

To avoid this conclusion it would have to be demonstrated that the means of class 2 systematic error populations (so-called) were themselves class 3 random variables appearing with different frequencies in some physical population sampled by the experiment. Then it would arguably be appropriate to convolute the distribution of the mean with that representing the purely random variation of the so-called systematic error to yield a frequency-based class 3 resultant distribution as required by the randomatic theory. But, the view that means (systematic errors proper) are distributed in the sense of appearing with different frequencies in some physical population would betray a misconceived identification of the relevant experiment and population. It has been noted that complete experimental specifications must identify, albeit implicitly, a particular so-called systematic error population as an essential feature of the experiment. While the corresponding distribution and mean are not known, they are determined through the definition of the experiment and not through some external random trial. Different workers independently following exactly the same experimental specification will therefore sample the same error population, producing results with a random variation, but all sharing the same bias from the true value. Thus the mean of the error is clearly sampled from a population of just one value. Since experimenters are interested in estimating the maximum possible or conceivable error in a *particular* specified experiment, the conservative is right to regard the mean as an undistributed quantity or as being “distributed” as a delta function at some unknown location. Of course, if the error population were investigated statistically, an estimate for its mean could be obtained and the error in the estimate characterized by a random distribution, The mean would be corrected for and the error in the mean would be treatable as random. However, the mean would then, by definition, not be a systematic error, but a measured quantity.

The randomaticist, if he seriously invokes a frequency-based distribution for the uninvestigated mean, is implying that it is in a literal sense singly sampled from some wider population once-and-for-all on behalf of all the independent experimental repetitions which could ever be conducted. Perhaps the population envisaged would be that of systematic errors, positive and negative, encountered in experiments in general, with the credible or maximum bounds re-scaled and re-dimensioned in each case to match those of the experiment in hand. Apart from the problematic question of whether this “super-population” of means itself has a zero mean, there would be no objection to randomatic procedures if this experiment were like that for which an uncertainty is required. But it is quite different from the conception of the experiment normally held. If *this* experiment were repeated, there would be a grand prefatory sampling of the error mean on each occasion followed by repetitions of the experiment as normally conceived. The results and the errors would then be different from those of the experiment for which an uncertainty is sought.

The constancy of the unknown mean which the conservative takes so seriously is therefore of quite a different nature from that of the determined outcome of a prefatory single sampling. It is built into the common concept of an experiment as a definite specified trial. As such there is no frequency-based rationale for treating uninvestigated systematic error means statistically and any justification of randomatic principles must hang on subjectivist arguments.

### 3.3 Justification 3: Randomatic Theory via Subjectivist Statistical Distributions

Modern subjectivist statisticians frequently identify probabilities with rational “degrees of belief.” Their general method is 1) to assign prior probabilities *p*(*x_j_*) to the possible results *x_j_*, of some trial reflecting their beliefs prior to making observations, and 2) to modify these in the light of evidence *E* (observed frequencies) using Bayes’ theorem to yield posterior probabilities:

$$p(x_{j}|E)=p(\;E|x_{j})p(x_{j})/\sum p(\;E|x_{k})p(x_{k})$$

In this way posterior values converge with those evaluated conventionally and they “realize” the same distributions as others. These will of course be different in general from their prior distributions.

In many subjectivist treatments of statistics, the psychological concept of a “degree of belier” is defined in terms of the betting odds which a subject would just be prepared to accept on such-and-such being the case. This notion, together with certain weak rationality constraints, for example betting in such a way as to avoid becoming the victim of a Dutch book, are held to be sufficient for deriving the axioms of probability theory.^5^

A familiar example of intuitive subjectivist practice is afforded by the situation where an experimenter has no information on whether a so-called systematic error is positive or negative and knows nothing about its magnitude except that it cannot exceed |*a*|. Having no reason to believe any value in the range *±a* more or less probable than another, he invokes a uniform Laplacian distribution of magnitude 1/2*a* between *±a.* Such a distribution is of course of class 3.

It has already been noted that it is essential to the randomatic theory that any distribution used to calculate uncertainties is of class 3. This is because a standard deviation, the only recognized “measure” of uncertainty, is defined relative to its distribution mean and so cannot reflect uncertainty arising from an unknown and unobservable non-zero distribution mean. (Covariances too, like those invoked in box 1, are defined relative to means and so can only allow for bias arising from correlation between purely random components of errors.) Unlike frequency-based distributions for systematic errors, subjectivist distributions are generally of class 3 because the sign of a systematic error is typically unknown. Where the distribution is not of class 3 corrections are sometimes applied to make it so. That subjectivist distributions are of class 3 is the great strength of Justification 3 compared to Justification 2.

However, because class 3 prior distributions which are not frequency-based are by definition undetermined by evidence, they are not objective. Different subjectivists will invoke different prior distributions and so calculate inconsistent standard deviations and uncertainties. More importantly, they will disagree in general from those which could be realized by repetition of the relevant trial. If they were to agree, it would be because there was sufficient knowledge to calculate approximate frequencies in advance. But then frequency-based distributions would have been invoked which are not generally of class 3 (cf. the preceding section). Randomaticists sometimes depend on class 3 subjectivist distributions to justify their recommended procedures for calculating uncertainties, but then make the incorrect assumption that their uncertainties are objective as they would be had their invoked distributions been frequency-based.

The lack of objectivity of prior distributions appears to some to be a fatal flaw in subjectivist statistics. In contrast, subjectivists see it as no great problem. They accept nowadays that such prior distributions are often non-objective best guesses or unbiased starting points for a Bayesian inference and are prepared to engage in mathematical analyses of “robustness” with respect to their uncertain features (roughly, how insensitive the posterior distribution is to any lack of realism in them) [e.g., 33].^6^ Objectivity is achieved through evidence and the process of Bayesian conditionalization. Unfortunately systematic error distributions are by their nature never investigated statistically and conditionalized. In this respect error theory differs crucially from other fields of statistical practice where Bayesian methods are employed. *Thus even subjectivists would regard subjectivist prior error distributions and a randomatic theory based on them as non-objective.*

With the failure of this justification, it is seen that in spite of its attractive features the randomatic theory lacks an objective foundation. Moreover, if uncertainties are defined to be maximum possible or conceivable errors in the results of particular specified experiments, the lack of objectivity can be expected to result in underestimated uncertainties on occasions. For example, two equal systematic uncertainties *±a* would combine to yield a resultant uncertainty of *±2a.* By treating the corresponding errors as being normally distributed, say, on the grounds that smaller errors are generally to be expected more often than larger ones, and associating the credible bounds *±a* with some confidence level close to one, the randomaticist will calculate an uncertainty corresponding to the resultant error of ± 1.41a at the same level of confidence. Under unfavorable circumstances both combining errors could be close to the same bound so that their resultant would virtually always lie outside the randomatic uncertainties. Although some experimenters have a compelling intuition that such unfavorable occurrences are “improbable,” especially where larger numbers of systematic errors combine, it is impossible to provide a physical rationale for this if systematic errors cannot be random variables in any objective sense. After all, none betting on the joint outcome of several particular dice known to have various undetermined biases would assume that the biases had zero expectations unless, unlike systematic errors, they had been randomly selected from a population in which this was true. The psychological origin of the intuition is no doubt the desire to believe that probabilities will always provide a basis for rational inference and action in the face of uncertainty, (cf. Fisher [35]). But if the preceding arguments are correct, this would appear not to be so.

## 4. Conclusions and Recommendations

It has been argued that the distinction between random and systematic errors is, when properly formulated, a clear and objective one applicable to all error populations. Moreover, which category an error falls into should determine whether it is treated statistically or not. Thus there is no room for a different fundamental distinction between error types A and B based on the method of treatment employed in given cases (cf. box 1). Frequently, conservatives have automatically taken so-called systematic errors like those of box 4 to be entirely systematic when they have in fact contained an assessable random component. Conversely, randomaticists have implicitly assumed that all so-called systematic errors are class 3 random errors having zero means. Thus both typical conservative and randomatic practices are based on unrealistic principles.

Given that many so-called systematic errors do contain both a random and systematic component, how are their corresponding uncertainties to be assessed? The formal answer to this question has already been given in section 2.2 where a procedure was recommended for estimating the uncertainty corresponding to a class 2 error (cf. fig. 2). However, this procedure is very often difficult to apply because insufficient information is available to characterize separately the class 1 and class 3 components of class 2 errors.

In dealing with such cases, experimenters of all persuasions often feel able to judge maximum possible or credible bounds beyond which the unknown distribution is certain to cover zero or a negligible probability. The bounds will often be symmetrical about zero, but it should not be supposed that they correspond to equal confidence intervals of the real distribution or license the experimenter to invoke a symmetrical or other class 3 distribution spanning them. As they are estimated from the worst possible or conceivable combination of physical effects it may well be that the incidental physical influences on the parent error population cause one or both tails of the distribution to become negligible well within their respective bounds (cf. the three curves shown in fig. 2). Thus many distributions are consistent with the choice of bounds and the mean of the real distribution could in principle lie anywhere between them.

Typically the experimenter will be unable to partition his uncertainty as defined by the bounds exactly into random and systematic components. Guided by the definition of uncertainty as maximum possible or conceivable error, the rigorous worker will adopt as the basis of further calculations a model derived from the maximum apportionment of uncertainty to the systematic category judged possible or conceivable. This is because resultant uncertainties calculated on the basis of an overestimated random component would be too small as random components combine in quadrature rather than additively. His judgment of the maximum apportionment of uncertainty to the systematic category will require him to assess the maximum range in which the mean of the actual distribution could lie. Thus, just as credible bounds were initially placed on the so-called systematic error distribution itself, so narrower credible bounds are placed on its mean, the systematic error as properly defined. Like the outer bounds, the inner bounds will not correspond to confidence limits and do not confine the mean in that sense; there is no pre-existing statistical sample to enable an estimate of the mean and the standard deviation of the estimate to be made. (If there were the mean would be like a measured quantity and it would be proper to correct for it and treat the remaining component as a class 3 random error). However, much vaguer information, perhaps that *in the given experimental context* smaller errors are more common than larger ones, can sometimes justify restricting the range of possible values of the mean. Where the information required for this is lacking, the inner credible bounds will become coincident with the outer which then become the systematic uncertainties they have so often been taken to be.

It may at first sight be thought that making such judgments is hopelessly arbitrary and the problems have to be acknowledged. But experimenters usually design their experiments so that the difficult uncertainties to evaluate are the least significant. Then some inaccuracy in judgments is tolerable. The difficulties are in any case largely common to all theories of error: judgments of maximum limits or credible bounds for the conservative and of systematic standard deviations for the randomaticist are often arbitrary in problem cases. However, their simple rules of procedure only disguise the difficulties without removing them. The approach recommended above brings them into the light and, while it calls for an additional judgment apportioning uncertainty between random and systematic categories, this is not markedly more difficult than those already required. More fundamentally, if the approach is realistic, as has been argued, it should be physically correct.

With these points in mind the following procedures are recommended for the estimation of experimental uncertainty.

### Recommendations for the Evaluation of Experimental Uncertainties

**1** The *whole* experiment should be defined (cf. section 2.3). All measurements, corrections, calibrations, external results, and single random samplings contributing to the final result of the experiment should be listed. All significant sources of error in the experimenter’s own part of the whole experiment should be identified. The nature and magnitude of uncertainties in all other results should be ascertained.

**2** Choose a confidence level (e.g., 95 or 99% probability) beyond which possibilities are regarded as being inconceivable. This level should be clearly stated.

**3** Decide to which class, 1 to 4, each error belongs. This decision should be made irrespective of whether the measurement or trial was actually repeated or not; the definition of these classes is in terms of what *would* be observed on repetition (cf. section 2.1). Where measurements have not been repeated, it should be possible to identify the class of any error from the specification of the measurement concerned giving the nominal conditions and procedures required for its execution and their permitted variations. In the case of single trials associated with an experiment (e.g., the selection of an incompletely characterized instrument or material) relevant error populations should be identified and one chosen as a basis for uncertainty estimation which minimizes the uncertainty (cf. section 3.2).

**4** If some subsidiary result of the experiment is observed to be subject to a significant class 4 error (so introducing a class 4 error into other results which it is used to calculate), attempt to identify the weak aspect of the control of the experiment which allowed it to occur. This may be done by experimental tests or by analysis of the experimental specification or both. When identified, repeat the experiment with better control if practicable. Alternatively, estimate the maximum range of values which the uncontrolled causative condition or conditions could possibly or conceivably take, and use these to compute maximum possible or credible errors in the quantities concerned. Treat these as systematic uncertainties according to the procedure of paragraph 5. If the source of the class 4 error cannot be identified, then of course no final uncertainty may be calculated.

**5** Estimate the maximum possible or credible absolute values in each direction of the class, 1 errors and of the constant components of the class 2 errors. Again, this may be done by reference to presumed measurement specifications or identified pre-existing error populations. Multiply each uncertainty by the coefficient ∂*y/*∂*x_j_* in the law of error propagation to obtain corresponding uncertainty components in the final result of the experiment. Add these together to obtain an overall systematic uncertainty in the final result.

**6** Identify those class 2 and 3 sources of random error which contribute directly to the final result. Multiply the observed or estimated standard deviation of each by the coefficient ∂*y/*∂*x_j_* to obtain corresponding components for the final result. Combine these in quadrature to yield a standard deviation for its random component of error.

**7** Having observed or inferred the form of the random component of error in the final result of the experiment, use the systematic uncertainties of paragraph 5 to define upper and lower limits for its mean, μ_Δ_*_y_*_max_ and −μ_Δ_*_y_*_min_ thus obtaining two “worst-case” distributions. Use the confidence level of paragraph 2 to calculate corresponding uncertainties, *u*_+_ and *u*_−_, according to the procedure of section 2.2 (see fig. 2). These overall uncertainties should be quoted together with 1) their systematic components, 2) their common random component, 3) the confidence level, and 4) any useful additional statistical information e.g., the number of degrees of freedom in calculated means or fitted curves (cf. Campion et al. [10]).

## Footnotes

2 Editor’s note: As the author readily acknowledges, error theory is a controversial field and his views are at odds with some recent thinking on uncertainty evaluation intended to secure international consensus. Yet it is believed that this paper is a valuable contribution to the literature on measurement uncertainties presenting a closely argued case for one of the standard positions on the subject. An alternative view will be presented by R. Collé in a subsequent issue of this Journal.

3 Such a resolution is always possible for any class 2 error Δx. Setting Δx=μ_Δx_+Δr, where μ_Δx_≡E(Δx) and the random variable Δr≡Δx − μ_Δx_ one has for its expected value E(Δx)=μ_Δx_+E(Δr). Thus E(Δr) = μ_Δx_− μ_Δx_=0 so that Δx=μ_Δx_ + Δr, Δr being a class 3 error as asserted.

4 Systematic error defined in terms of a class 1 error or error components ≡μ_Δ_*_y_*≡*E*(Δ*y*)≡*E*(*y* – *y*_0_)*=E*(*y*)−*y*_0_≡systematic error defined in terms of bias.

5 The most detailed foundational development of subjectivist theory in terms of rational betting and Bayesian conditionalization has been undertaken by de Finetti [24,25]. Useful reviews of his work may be found in Gillies [26,27]. Clear statements of subjectivist ideas have also been presented by Savage [28,29] who in the latter gives a schematic subjectivist account of a physical measurement (the weighing of a potato). Two wide-ranging collections of subjectivist and Bayesian papers are “Studies in Subjective Probability.” edited by Kyberg and Smokler [30], and “Bayesian Analysis in Econometrics and Statistics.” edited by Zellner [31]. A modern mathematical text extending the tradition in a philosophically conscious way is “Statistical Decision Theory” by Berger [32]. A well-known critique of subjectivist methodology was presented by Fisher in “Statistical Methods and Scientific Inference.”[1]

6 Early subjectivists sometimes regarded Bayes’ postulate or some variant thereof as an unchallengable axiom; this view led to well-known logical difficulties and is not generally held by modern subjectivists [34].
